# Respiratory virus surveillance in hospitalised pneumonia patients on the Thailand-Myanmar border

**DOI:** 10.1186/1471-2334-13-434

**Published:** 2013-09-16

**Authors:** Paul Turner, Claudia Turner, Wanitda Watthanaworawit, Verena Carrara, Naw Cicelia, Carole Deglise, Christina Phares, Luis Ortega, Francois Nosten

**Affiliations:** 1 Shoklo Malaria Research Unit, Mae Sot, Thailand; 2 Mahidol-Oxford Tropical Medicine Research Unit, Bangkok, Thailand; 3 Centre for Tropical Medicine, University of Oxford, Oxford, UK; 4 Première Urgence–Aide Médicale Internationale, Mae Sot, Thailand; 5 Centers for Disease Control and Prevention Collaboration, Atlanta, GA, USA

**Keywords:** Pneumonia, Refugee, Influenza, Respiratory, Virus

## Abstract

**Background:**

Pneumonia is a significant cause of morbidity and mortality in the developing world. Viruses contribute significantly to pneumonia burden, although data for low-income and tropical countries are scarce. The aim of this laboratory-enhanced, hospital-based surveillance was to characterise the epidemiology of respiratory virus infections among refugees living on the Thailand-Myanmar border.

**Methods:**

Maela camp provides shelter for ~45,000 refugees. Inside the camp, a humanitarian organisation provides free hospital care in a 158-bed inpatient department (IPD). Between 1st April 2009 and 30th September 2011, all patients admitted to the IPD with a clinical diagnosis of pneumonia were invited to participate. Clinical symptoms and signs were recorded and a nasopharyngeal aspirate (NPA) collected. NPAs were tested for adenoviruses, human metapneumovirus (hMPV), influenza A & B, and RSV by PCR.

**Results:**

Seven hundred eight patient episodes (698 patients) diagnosed as pneumonia during the enhanced surveillance period were included in this analysis. The median patient age was 1 year (range: < 1-70), and 90.4% were aged < 5 years. At least one virus was detected in 53.7% (380/708) of episodes. Virus detection was more common in children aged < 5 years old (<1 year: OR 2.0, 95% CI 1.2-3.4, *p* = 0.01; 1-4 years: OR 1.4, 95% CI 0.8-2.3, *p* = 0.2). RSV was detected in 176/708 (24.9%); an adenovirus in 133/708 (18.8%); an influenza virus in 68/708 (9.6%); and hMPV in 33/708 (4.7%). Twenty-eight episodes of multiple viral infections were identified, most commonly adenovirus plus another virus. RSV was more likely to be detected in children <5 years (OR 12.3, 95% CI 3.0-50.8, *p* = 0.001) and influenza viruses in patients ≥5 years (OR 2.8, 95% CI 1.5-5.4, *p* = 0.002). IPD treatment was documented in 702/708 cases; all but one patient received antimicrobials, most commonly a beta-lactam (amoxicillin/ampicillin +/−gentamicin in 664/701, 94.7%).

**Conclusions:**

Viral nucleic acid was identified in the nasopharynx in half the patients admitted with clinically diagnosed pneumonia. Development of immunisations targeting common respiratory viruses is likely to reduce the incidence of pneumonia in children living refugee camps and similar settings.

## Background

Pneumonia remains a leading cause of mortality globally: 4.2 million pneumonia deaths were recorded in 2004 [1]. The highest incidence of disease occurs in young children [2,3]. An estimated 156 million pneumonia episodes occur annually in children less than five years old. Over 95% of these occur in the developing world, where the incidence of clinical pneumonia is estimated to be 0.29 episodes per child-year. Almost three-quarters of childhood pneumonia deaths occur in sub-Saharan Africa and Southeast Asia [4]. Bacterial pathogens, most notably *Streptococcus pneumoniae* and *Haemophilus influenzae* type B, are important vaccine-preventable causes of pneumonia [5]. Viruses, in particular influenza and respiratory syncytial virus (RSV), are also responsible for a large number of pneumonia cases each year [6]. Using global population data for 2005, for children under the age of five years, it was estimated that RSV was responsible for over 30 million episodes of lower respiratory tract infections (LRTI), with ~3 million of these requiring hospital admission, and 66,000-199,000 deaths [7]. By similar analyses of data from 2008, influenza viruses were estimated to cause 20 million LRTI and 1 million severe LRTI, with 28,000-111,500 deaths, in children aged less than five years [8]. In both of these reviews, 99% of deaths from either influenza–or RSV–associated LRTI occurred in the developing world. These viruses may be responsible for up to 35% of LRTI (RSV 22% [7] and influenza 13% [8]) in children under the age of five years. Other viral pathogens associated with childhood pneumonia include adenoviruses, human metapneumovirus (hMPV), and parainfluenza viruses [5,9].

Approximately one-third of the worldwide refugee population of 15 million live in camps [10]. These camps are often crowded with poor sanitation, providing ideal conditions for transmission of respiratory pathogens [11,12]. There have been refugees from Myanmar (Burma) living in camps in Thailand since 1984. In 2006, lower respiratory tract infections were estimated to be the cause of 9% of deaths, and were responsible for 25% of all reported morbidity, in the under-5 age group of the border refugee population. The overall under-5 year mortality rate was 6 per 1,000, giving an estimated LRTI-specific mortality rate of 0.5 per 1,000 [13].

In 2007, the US Centers for Disease Control and Prevention (CDC) and the Shoklo Malaria Research Unit (SMRU) established a respiratory virus surveillance programme in the Burmese refugee population living in Maela camp, Northwest Thailand. The programme included patients admitted to hospital with pneumonia during April 2009-September 2011. The aim of in-patient surveillance was to determine the relative burden of virus-associated pneumonia. The results of 30 months of in-patient surveillance are presented here.

## Methods

### Site and population

Maela camp is located in rural Tak province approximately 500 km from Bangkok. It is the largest of the nine camps on the Thailand-Myanmar border, housing approximately 45,000 people in a 4 km^2^ area, and has been in continuous operation since 1984. Karen is the predominant ethnicity in the camp population. General healthcare is provided by the non-governmental organisation Première Urgence–Aide Médicale Internationale (PU-AMI). Camp residents receive World Health Organisation (WHO) Expanded Programme on Immunisation (EPI) immunisations, but immunisations against respiratory pathogens (*Haemophilus influenzae* type B, influenza viruses, and *Streptococcus pneumoniae*) are not available.

### Enrolment and data collection

From April 2009 to September 2011, laboratory-enhanced respiratory surveillance was undertaken at the in-patient department (IPD) of the Maela PU-AMI hospital. Throughout this period, trained local health workers reviewed IPD admission logs on six days each week to identify patients with an admission diagnosis of pneumonia, including those who were admitted on the seventh day. Health workers invited all pneumonia patients to participate in enhanced surveillance and enrolled all who agreed. For enrolled patients, health workers completed a brief symptoms questionnaire by patient interview and record review, and collected nasopharyngeal aspirates (NPA) as previously described [14].

Patient episodes were subsequently excluded from analyses if they (a) failed to meet the surveillance case definition for pneumonia (Table 1), (b) occurred within 14 days of previous episode in the same patient, or (c) lacked adequate laboratory specimens. Surveillance case definitions were based on those devised by WHO for children under five years of age [15,16]. For older individuals, in whom a satisfactory clinical case definition is lacking, the surveillance case definition was based on that described by the British Thoracic Society [17].

**Table 1:** Pneumonia case definitions

**Age group**	**Definition**	
< 5 years	*Pneumonia*	Cough OR difficulty breathing AND Increased respiratory rate*
	*Severe pneumonia*	Cough OR difficulty breathing AND At least one of: Lower chest wall indrawing; nasal flaring; grunting
	*Very severe pneumonia*	Cough OR difficulty breathing AND At least one of: Central cyanosis; inability to feed or vomiting everything; convulsions, lethargy, or unconsciousness
≥ 5 years		Fever ≥ 38°C OR history of fever AND Cough OR difficulty breathing AND Abnormal chest examination (e.g. crepitations, asymmetric breath sounds, or dullness to percussion)

* Measured over one minute: > 60 if < 2 months of age; > 50 if 2-11 months of age; > 40 if 11-59 months of age.

### Laboratory methods

NPA specimens, in 1 ml viral transport medium (VTM, prepared in-house), were transported daily to the SMRU microbiology laboratory, which is located in the town of Mae Sot, approximately 50 km from Maela. Specimens were placed into an insulated cool box immediately after collection and were transported back to the Mae Sot laboratory within eight hours of collection, where they were stored at−80°C until analysis.

Viral nucleic acid was extracted from thawed NPA-VTM specimens using commercial kits, following the manufacturer’s instructions (QIAamp Viral RNA minikit [Qiagen, Hilden, Germany], April 2009 until September 2010; viral nucleic acid extraction protocol of the MagCore HF16 automated extractor [RBC Bioscience, Taiwan], October 2010 until September 2011). Extracts were analysed by real-time reverse-transcription PCR (rRT-PCR) for adenoviruses, hMPV, influenza viruses (A and B, with typing of influenza A viruses to detect seasonal H1/H3 and pandemic H1 strains), and RSV as described elsewhere [14,18,19]. An internal control human RNaseP PCR was included to confirm the specimen adequacy and to identify PCR inhibition [20]. Specimens were considered positive if a virus PCR cT value was < 40 with appropriate run control results. To ensure the reproducibility of results approaching the assay limits of detection (approximately 100 copies per reaction for each target; SMRU internal QC data), specimens with low positive PCR results (cT values of 35-39) were repeated and only if the cT was < 40 in both runs was the virus PCR considered positive.

### Data management and analysis

Clinical and laboratory data were recorded on paper-based case record forms and subsequently entered into an Access 2003 database (Microsoft, Redmond, WA, USA) and systematically checked for errors by comparison with the original case record forms. Data were analysed by Stata/IC version 12.1 (StataCorp, College Station, TX, USA).

Proportions were analysed by chi-squared or Fisher’s exact tests as appropriate. Logistic regression was used to calculate odds ratios (OR) and their 95% confidence intervals (CI). Multivariate models were constructed to determine relationships between age, viral detection, pneumonia severity (<5 years old only), and antimicrobial use prior to admission. Two-tailed *p-*values of < 0.05 were considered significant.

### Ethics

This surveillance program underwent ethical and regulatory review at CDC, and was determined not to meet the definition of research. Local ethical review in Maela was not possible at the commencement of surveillance. However, the surveillance activity was discussed with PU-AMI staff, and all concerns were addressed, prior to the beginning of the project. Verbal consent was obtained from each potential participant, or their parent/legal guardian in the case of children aged <15 years, prior to enrolment in the surveillance programme.

## Results

Among all IPD pneumonia admissions, 835 patient episodes were enrolled in enhanced surveillance. After review of the symptom questionnaire, 117 patient episodes were excluded because of failure to meet the case definition and one episode was excluded for a patient who presented twice within 14 days. In three episodes, NPA specimens were not collected and in another six, the specimens were technically inadequate (internal control PCR negative). The remaining 708 patient episodes were included in the following analyses.

### Patient characteristics

A total of 698 individuals were sampled (689 patients with single episodes, eight patients with two episodes, and one with three episodes). The median age at presentation was one year (IQR < 1-2; range < 1 to 70). Six hundred and forty patients (90.4%) were aged < 5 years (Table 2). There were significantly more males in the < 1 year age group compared with the older patients (64.7% vs. 52.2%, *p* = 0.001). Patients presented at a median of 4 days after symptom onset (IQR 2-6). Eighty five percent of pneumonia episodes in the patients aged < 5 years were classified as severe or very severe; this did not vary by gender or the duration of illness prior to admission (data not shown). Children aged 1-4 years were less likely to have severe or very severe pneumonia compared with those aged < 1 year (OR 0.6, 95% CI 0.4-0.9, *p* = 0.03). Treatment was documented for 702/708 (99.2%) episodes: all but one patient received an antimicrobial drug, most commonly amoxicillin or ampicillin +/−gentamicin (664/702, 94.6%).

**Table 2:** Patient demographic details and virus prevalence

**Characteristic**	**Age group**		
	**< 1 year**	**1-4 years**	**≥ 5 years**
Pneumonia episodes, N (%)	289	351	68
*Non-severe*	33 (11.4)	62 (17.7)	68 (100)
*Severe / Very severe*	256 (88.6)	289 (82.3)	-
Gender, N (%)			
*Female*	102 (35.3)	168 (48.3)	31 (45.6)
*Male*	187 (64.7)	180 (51.7)	37 (54.4)
Days unwell at admission review, median (IQR)	4 (3-7)	3 (2-6)	4 (3-7)
Antimicrobial use ≤ 14d before admission, N (%)	133 (46.5)	129 (37.0)	22 (33.3)
Any virus detected, N (%)	173 (59.9)	178 (50.7)	29 (42.7)
*Adenovirus*	39 (13.5)	80 (22.8)	14 (20.6)
*hMPV*	17 (5.9)	15 (4.3)	1 (1.5)
*Influenza A or B*	19 (6.6)	35 (10.0)	14 (20.6)
*RSV*	113 (39.1)	61 (17.4)	2 (2.9)
*Multiple viruses detected*	14 (4.8)	12 (3.4)	2 (2.9)

### Virus detection

Viral nucleic acid was detected in 53.7% (380/708) NPA specimens. The rank order of detection was RSV (176, 24.9% of NPA), adenovirus (133, 18.8%), influenza A (58, 8.2%), hMPV (33, 4.7%), and influenza B (10, 1.4%).

**Figure 1:**
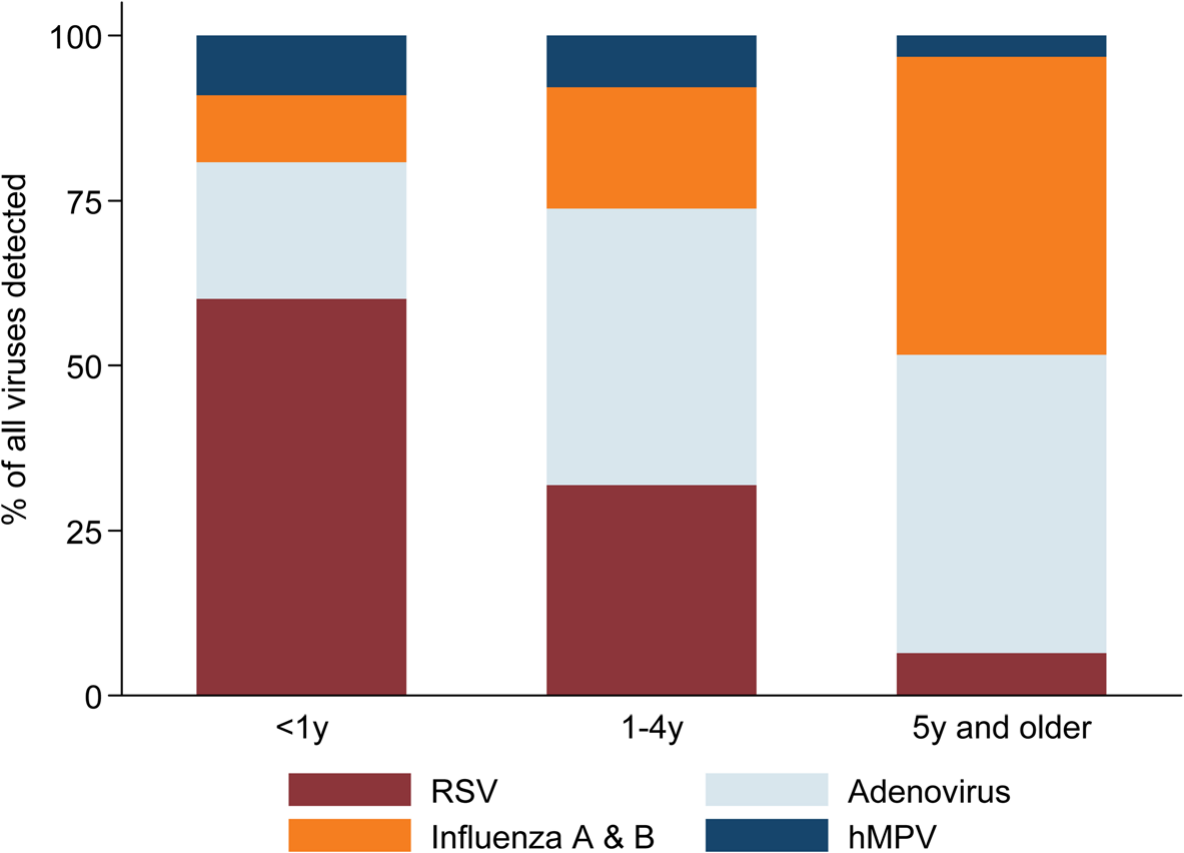
**Viral detection by age group.** Detection of individual viruses as a proportion of all viruses detected in patients of each age group.

Detection of viruses varied considerably by age (Table 2). Patients aged < 5 years were more likely to have viral nucleic acid detected in the NPA compared with those five years or older (< 1 year old: OR 2.0, 95% CI 1.2-3.4, *p* = 0.01; 1-4 years old: OR 1.4, 95% CI 0.8-2.3, *p* = 0.2). RSV was significantly more likely to be detected in those patients presenting at < 5 years of age (< 1 year old: OR 21.2, 95% CI 5.1-88.2, *p* < 0.001; 1-4 years old: OR 6.9, 95% CI 1.7-29.1, *p* = 0.008). The same trend was seen for hMPV, although not statistically significant as a result of the small numbers of infections detected in older children and adults (< 1 year old: OR 4.2, 95% CI 0.5-32.0, *p* = 0.2; 1-4 years old: OR 3.0, 95% CI 0.4-23.0, *p* = 0.3). Influenza viruses were more commonly detected in older children and adults compared with those aged less than one year (1-4 years old: OR 1.6, 95% CI 0.9-2.8, *p* = 0.1; ≥ 5 years old: OR 3.7, 95% CI 1.7-7.8, *p* = 0.001). Adenoviruses were also more likely to be detected in those aged one year or more (1-4 years old: OR 1.9, 95% CI 1.2-2.9, *p* = 0.003; ≥ 5 years old: OR 1.7, 95% CI 0.8-3.3, *p* = 0.1) (Figure 1).

Diagnosis of severe or very severe pneumonia in the < 5 year age group was associated with detection of RSV (OR 1.9, 95% CI 1.1-3.2, *p* = 0.03). The trend remained the same, although the association became statistically non-significant, when controlling for age and detection of other viruses in a multivariate logistic regression model (AOR 1.5, 95% CI 0.8-2.7, *p* = 0.2). In the same multivariate model, detection of adenovirus (AOR 0.5, 95% CI 0.3-0.9, *p* = 0.01) or an influenza virus (AOR 0.5, 95% CI 0.3-1.0, *p* = 0.05) were associated with a lower likelihood of severe or very severe pneumonia diagnosis.

Multiple viruses were detected in 4.0% (28/708) specimens. Two viruses were detected in 26 specimens (13 adenovirus + RSV; 8 influenza + RSV; 3 adenovirus plus influenza; 2 adenovirus + hMPV) and three viruses were detected in two specimens (1 adenovirus + influenza + hMPV; 1 adenovirus + influenza + RSV). There were no associations between multiple virus detection with age or severity of pneumonia (data not shown).

**Figure 2:**
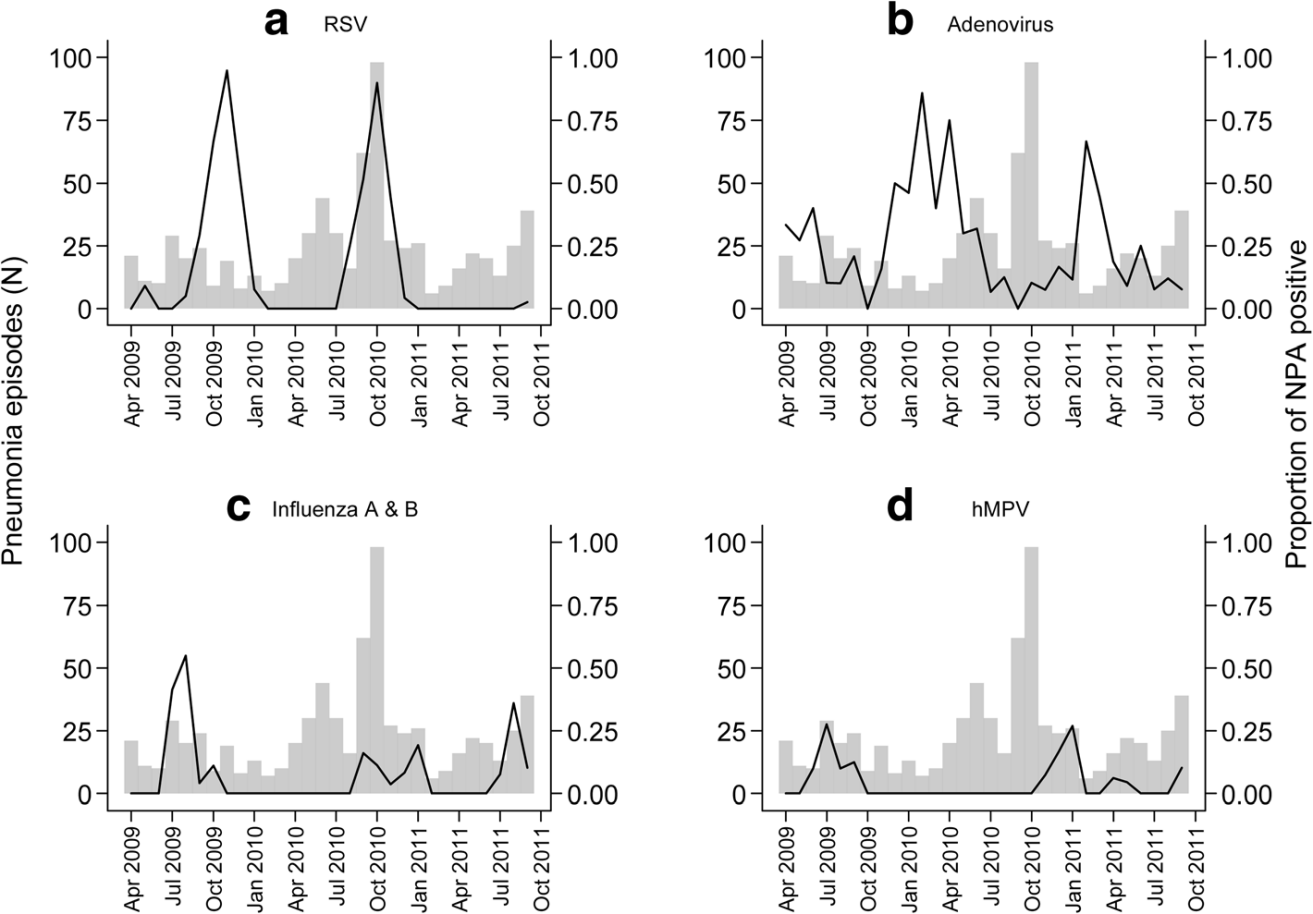
**Seasonality of virus detection, April 2009-September 2011.** Lines indicate the proportion of nasopharyngeal aspirate specimens positive for the virus by month: **(a)** RSV, **(b)** Adenovirus, **(c)** Influenza A & B, and **(d)** hMPV. The bars indicate the number of pneumonia episodes sampled each month.

Virus detection varied by season. RSV, influenza viruses, and hMPV were all detected in the wet (June–October) and cool (November–February) seasons, whereas adenovirus detection occurred year round and peaked in the late cold and hot (March–May) seasons (Figure 2).

Interestingly, in an age-adjusted analysis, patients who had received an antimicrobial in the two weeks preceding admission were more likely to be RSV PCR positive (AOR 1.7, 95% CI 1.2-2.5, *p* = 0.003). No such association was seen for the other viruses.

### Influenza virus dynamics

**Figure 3:**
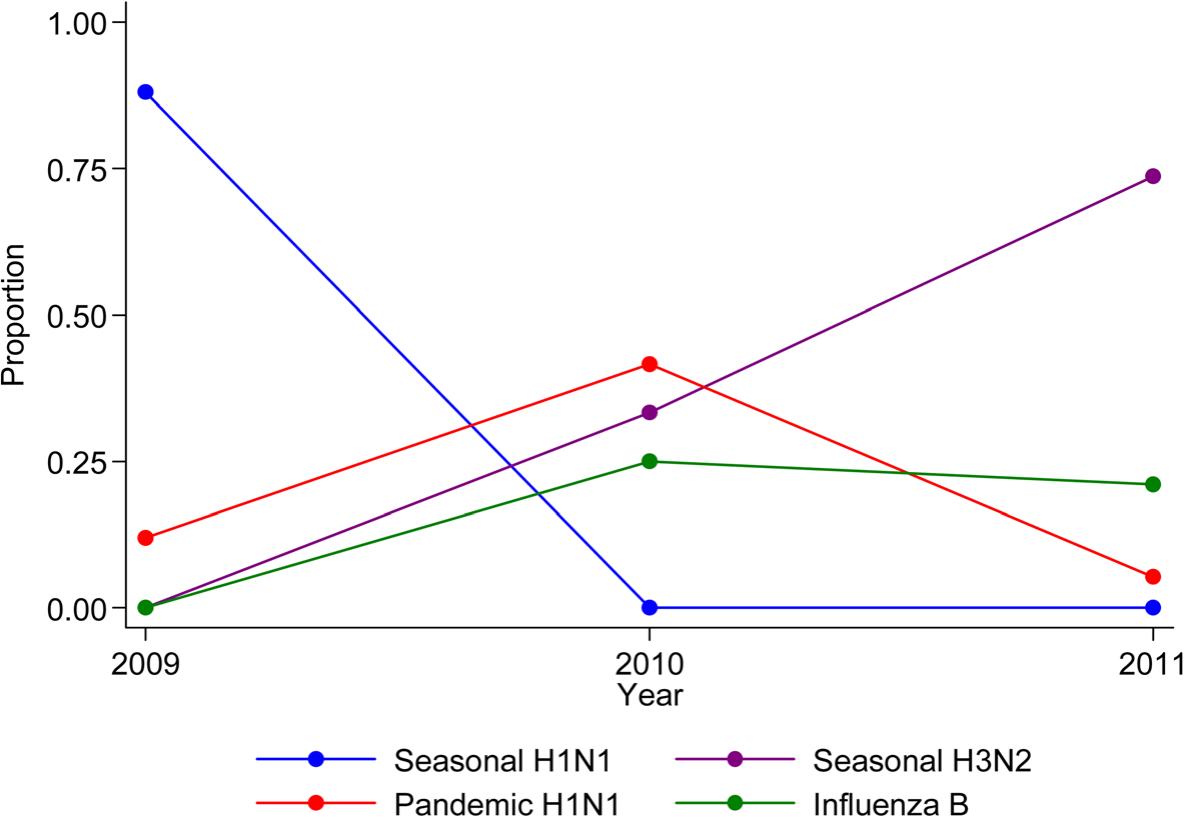
**Influenza virus dynamics by year.** The individual lines represent the relative proportions of influenza A sub-types (seasonal H1N1/H3N2 and pandemic H1N1) and influenza B detected by year of surveillance.

Seasonal influenza A (H1N1) accounted for 88.0% of all influenza virus detections in 2009 but was not identified in subsequent years. Influenza A (H3N2) detections increased from 0% in 2009 to 73.7% of influenza viruses in 2011. Pandemic influenza A (H1N1 2009) detections peaked in 2010 (41.7% of all influenza virus detections). Influenza B was not found in 2009, but in subsequent years accounted for up to 25.0% of influenza virus detections (Figure 3). Twenty five patients with influenza A associated pneumonia (admitted May–October 2009) have been described in detail in a previous manuscript [14].

## Discussion

Laboratory-enhanced surveillance has documented the contribution of respiratory viruses to 708 hospitalised clinical pneumonia episodes occurring in a crowded refugee camp on the Thailand-Myanmar border during April 2009 to September 2011. As expected, the vast majority of patients were aged less than five years [2]. Viruses were detected in the nasopharynx of half of the cases. Virus detection was significantly more likely in infants compared to older children and adults, although the number of adult cases was small. During the same period, routine hospital surveillance detected 2,081 in-patient LRTI episodes, including 32 (1.5%) deaths and 1,340 (64.4%) episodes in children under five years [21]. Therefore, IPD pneumonia episodes under enhanced surveillance reported here accounted for 34.0% of all IPD LRTI episodes (47.8% of IPD LRTI episodes in children aged less than five years and 9.2% of episodes in persons aged five years or older).

The Maela data add to the scant data on the aetiology of pneumonia in refugee populations. The results are broadly consistent with a similar surveillance programme conducted in two Kenyan refugee camps [22], where 51.3% patients with severe acute respiratory infection (SARI) had at least one of adenovirus, hMPV, influenza A/B, parainfluenza virus 1-3, or RSV detected. In the Kenya surveillance, adenovirus was the commonest virus detected (22.7% of specimens), followed by RSV (14.1%). An influenza virus was detected in 11.6% of specimens. The majority (82%) of hospitalised SARI cases were children less than five years of age.

Results from pneumonia aetiology studies from various locations in the developing world have confirmed the high prevalence of virus detection in hospitalised pneumonia episodes in young children [23,24]. RSV has been frequently identified as the commonest virus and is associated with severe infections [25-28]. A significant proportion of RSV infections occur in the first year of life, although both primary infection and re-infections are common in older children [26,29,30]. RSV has previously been documented to be a significant pathogen in Maela, with an incidence of RSV-associated pneumonia of 0.24 episodes per child-year at risk in a cohort of infants followed from birth until two years of age [31,32]. Our results confirm that RSV is the leading virus associated with pneumonia in the general Maela camp population. The majority of infections were in young children and only two (2/176, 1.1%) RSV infections occurred in patients aged five years or older.

Influenza viruses were detected in almost 10% of patient episodes of pneumonia. This figure is consistent with previously published data on influenza hospitalisations. Simmerman and Uyeki determined that influenza viruses were detected in 6-14% of hospitalised pneumonia cases in a recent review of East and Southeast Asian data [33].

The role of adenoviruses in the aetiology of pneumonia remains unclear. Frequent re-infection and persistence in young children makes their detection in NPA specimens at the time of pneumonia diagnosis difficult to interpret [34,35]. A recent case–control study from Kenya, undertaken as part of a multi-centre paediatric pneumonia aetiology study (PERCH), did not find a higher odds of adenovirus detection in pneumonia cases compared with controls [36]. In that study, only the detection of RSV in the upper respiratory tract had a significant association with hospitalisation for pneumonia.

WHO definitions for clinical pneumonia in childhood were used in our enhanced surveillance in Maela. These definitions were designed to have optimal sensitivity for the diagnosis of potentially life-threatening bacterial infection in resource-poor settings [15]. It was accepted that some over-diagnosis and unnecessary treatment would occur [37]. As was demonstrated in a South African study, where virus-associated pneumonias were reduced by a third in children given a nine-valent pneumococcal conjugate vaccine, a proportion of virus-positive individuals would have had a secondary bacterial infection [38], but many will have received an unnecessary course of antibiotics. Recent work from Pakistan has confirmed that placebo and amoxicillin had equivalent efficacy for WHO non-severe pneumonia and that oral amoxicillin at home is an acceptable alternative to hospital admission and parenteral antibiotics for severe pneumonia [39,40]. In Maela, almost all patients were treated with at least one antimicrobial drug, yet over a third (excluding adenovirus) had a proven viral infection. Collectively, these findings point to the likely over treatment of viral infections in children with WHO defined pneumonia, which may contribute to the rising prevalence of antimicrobial resistance in the developing world [41].

The enhanced surveillance system had several limitations. Not all hospitalised pneumonia episodes were captured and therefore it was not possible to calculate virus-specific incidence rates. Estimating the representativeness of the patients enrolled by comparison of enhanced surveillance results with routine surveillance figures is problematic. However, as we note, if a direct comparison is made, enhanced surveillance included 47.8% (640/1,340) of all LRTI episodes identified through routine surveillance in children under five years of age but only 9.2% (68/741) of episodes in patients aged five years or older. Differences in case definitions used in the routine surveillance compared with enhanced surveillance is the most likely explanation for this discrepancy [21]. For the under-5 age group the definitions were almost identical (i.e. WHO-based) but for the older age group LRTI was defined in routine surveillance by the presence of a fever plus cough or sore throat and shortness of breath/difficulty breathing. Absence of a requirement for abnormal chest signs likely resulted in inclusion of many non-pneumonia cases within the LRTI category. The low number of deaths recorded in the HIS system suggests that severity was not a significant reason for non-inclusion. Also, since PU-AMI hospital was the only general medical admissions unit within the camp, there were not significant numbers of patients missed as a result of admission to other hospitals. However, it remains possible that the older participants were not representative of the entire hospitalised pneumonia patient group. Additional severity data (e.g. need for supplemental oxygen and length of stay) and outcome data were not collected, which further limit the conclusions that can be drawn from the surveillance and the comparisons that can be made with other studies. The panel of viruses tested for included the key pathogens for which evidence of an association with pneumonia is proven, but inclusion of PCR assays to detect additional respiratory viruses would have added value and may have identified a higher prevalence of multiple infections. Studies using multiplexed virus PCR assays have detected both an increased proportion of children with single and multiple viral lower respiratory infections [42]. Both human bocavirus and rhinoviruses may be detected in a large proportion of pneumonia cases, although data regarding causality from case–control studies are still limited [36,43]. Despite this, it was demonstrated that influenza virus and/or RSV were associated with a third of hospitalised pneumonia episodes in Maela.

The cost of in-patient pneumonia treatment is high. In two recent studies, the estimated average cost per district hospital pneumonia admission was US$99.26 in Kenya and US$490.80 in Thailand [44,45]. Influenza infections are vaccine preventable. A vaccine to prevent RSV infection remains elusive, although progress continues to be made [46,47]. Inclusion of influenza immunisation, and RSV immunisation should it become available, in the immunisation programme for refugees on the Thailand-Myanmar border would likely significantly reduce the burden of pneumonia requiring hospitalisation.

## Conclusions

Viruses were commonly identified in Burmese refugees admitted to hospital with clinically-diagnosed pneumonia. Use of influenza immunisation and the development of vaccines targeting other common respiratory viruses would be likely to reduce the incidence of pneumonia in children living in refugee camps and similar settings.

## Competing interests

The authors declare that they have no competing interests.

## Authors’ contributions

PT, VC, CT, CD, CP, LO, and FN conceived the surveillance project. NC and CT were responsible for specimen and data collection. WW performed the laboratory work. PT and VC did the data analysis. PT prepared the first draft of the manuscript. All authors reviewed and contributed to revisions of the manuscript. All authors read and approved the final manuscript.

## Pre-publication history

The pre-publication history for this paper can be accessed here:

http://www.biomedcentral.com/1471-2334/13/434/prepub
